# The Predictive Value of Structural OCT in Preclinical AD

**DOI:** 10.1002/alz.090881

**Published:** 2025-01-09

**Authors:** Jessica Alber

**Affiliations:** ^1^ Butler Hospital Memory and Aging Program, Providence, RI, USA; George & Anne Ryan Institute for Neuroscience, Kingston, RI, USA; University of Rhode Island, Kingston, RI USA

## Abstract

**Background:**

Research shows that structural retinal changes, measured via spectral‐domain optical coherence tomography (SD‐OCT), are sensitive diagnostic biomarkers in AD. Our recent work aims to validate retinal biomarkers as risk biomarkers in preclinical AD. We compared retinal layer thickness to plasma ptau217 (a valid measure of Aβ pathophysiology in preclinical AD) to determine the predictive value of structural retinal changes in preclinical AD.

**Method:**

A total of 120 cognitively unimpaired (CU; CDR=0, MoCA>26) older adults aged 55‐80 underwent SD‐OCT imaging. Plasma samples were analyzed with the Simoa ALZpath pTau 217 assay. Multinomial logistic regression models, with age, APOE E4 status and first degree family history as covariates, were used to assess the predictive value of each retinal layer on plasma ptau217 concentrations, with emphasis on layers that have shown thinning in clinical AD (macular retinal nerve fiber layer, mRNFL; ganglion cell layer, GCL; inner plexiform layer, IPL). Additional iterative models added mRNFL, then GCL and IPL, to assess the combined predictive value of the retinal layers. ROC analysis with assay‐specific plasma ptau217 cut‐off .495 was used to determine how well each retinal layer and/or the retinal layers combined predicted preclinical AD status. Partial correlations (controlling for age) were performed to determine the relationships between individual retinal layer thicknesses and plasma ptau217.

**Result:**

There were significant positive correlations between the thickness of the inner inferior and superior GCL and IPL and plasma ptau217 (all p<.05). Inner inferior and superior mRNFL and GCL thickness were significant predictors of plasma ptau217. The model that had the best performance included age, APOE status, first degree family history, and the inner inferior and superior thickness of the mRNFL, GCL, and IPL, with AUC = .90, (95%CI = .86‐.93, p<.01).

**Conclusion:**

Retinal thickness, measured by SD‐OCT, could be a useful screening tool to identify those at high risk for preclinical AD who may benefit from more invasive or expensive neuroimaging for confirmation. Longitudinal follow‐up is ongoing to determine the prognostic value of SD‐OCT in predicting conversion to symptomatic AD.

